# Sulfate-compatible density marks an intratrimeric junction in the *Escherichia coli* dihydrolipoamide succinyltransferase catalytic domain

**DOI:** 10.1107/S2053230X2600909X

**Published:** 2026-09-17

**Authors:** Bison Lim, Eunji Kim, Jungeun Byun, Minjeong Kim, Hyo Jung Kim

**Affiliations:** a College of Pharmacy Woosuk University Wanju55338 Republic of Korea; Baylor College of Medicine, Houston, USA

**Keywords:** dihydrolipoamide succinyltransferase, 2-oxoglutarate dehydrogenase complex, *Ec*DSCD, *F*432 crystal structure, intratrimeric junction

## Abstract

The catalytic domain of *Escherichia coli* dihydrolipoamide succinyltransferase (*Ec*DSCD), the E2 component of the 2-oxoglutarate dehydrogenase complex, forms the oligomeric core of the multi-enzyme assembly. Here, we report the crystal structure of *Ec*DSCD determined in space group *F*432. The *F*432 crystal symmetry captures a compact trimeric building block and a symmetry-expanded 24-subunit assembly consistent with the octahedral architecture of the E2 catalytic core. Structural analysis revealed sulfate-compatible electron density near Lys196 at a crystallographic threefold intratrimeric junction. Comparison with previously reported *Ec*DSCD structures suggests that this density marks a structurally relevant oxyanion-sensitive hotspot at the intratrimeric junction. Notably, the α1-helix is positioned adjacent to the Lys196-centered junction and extends towards the active-site pocket, where it adopts a shifted orientation relative to other *Ec*DSCD crystal forms. These observations link sulfate-compatible density, the intratrimeric junction and α1-helix positioning within the *Ec*DSCD 24-mer, providing a structural framework for understanding local oxyanion-sensitive features in the E2 catalytic core.

## Abstract 2

The crystal structure of the catalytic domain of *E. coli* dihydrolipoamide succinyltransferase in space group *F*432 identifies a sulfate-compatible intratrimeric junction associated with Lys196 and α1-helix positioning near the active-site pocket.

## 1. Introduction

The *Escherichia coli* 2-oxoglutarate dehydrogenase complex (OGDHc) is a large multi-enzyme assembly that catalyzes the oxidative decarboxylation of 2-oxoglutarate and the transfer of the resulting succinyl group to coenzyme A (CoA). Similar to other α-ketoacid dehydrogenase complexes, OGDHc is composed of multiple copies of three enzymatic components: E1 (2-oxoglutarate dehydrogenase), E2 (dihydrolipoamide succinyltransferase) and E3 (dihydrolipoamide dehydro­genase) (Frank *et al.*, 2007 ▸; Reed, 2001 ▸). Among these components, E2 has a central architectural role, forming the inner scaffold of the complex while also catalyzing acyl transfer to CoA. The E2 component has a modular architecture composed of flexible N-terminal lipoyl-bearing regions, peripheral subunit-binding elements and a C-terminal catalytic domain (Ricaud *et al.*, 1996 ▸; Knapp *et al.*, 1998 ▸). The catalytic domain of the *E. coli* E2 component, referred to here as *Ec*DSCD, forms the inner core of the OGDHc E2 assembly. This domain assembles into trimers, and eight trimers further organize into an octahedral 24-mer particle with 432 symmetry (Knapp *et al.*, 2000 ▸). This high-symmetry architecture creates a stable catalytic platform and places active-site regions in the context of subunit interfaces. Therefore, the structural and catalytic organization of *Ec*DSCD is closely linked not only to the fold of individual protomers but also to the way that these protomers assemble into the higher order E2 core (Bliven *et al.*, 2018 ▸; Duarte *et al.*, 2022 ▸).

Previous structural studies of *Ec*DSCD and related E2 catalytic domains have established the general organization of the 24-mer core and revealed that the active-site environment is shaped by the intersubunit architecture (Andi *et al.*, 2019 ▸; Knapp *et al.*, 1998 ▸, 2000 ▸). The catalytic pocket is positioned near channels and interfaces formed between neighboring subunits, where local electrostatic features may influence substrate access and catalytic domain organization. Because CoA contains negatively charged phosphate groups, positively charged regions near the active-site entrance or subunit interfaces may contribute to the stabilization of anionic ligand moieties (Wang *et al.*, 2014 ▸; Mattevi *et al.*, 1992 ▸). In this context, phosphate-like oxyanions observed in crystal structures can provide useful markers of electrostatically favorable regions. Sulfate ions, although often derived from crystallization conditions, can occupy positively charged pockets and reveal local oxyanion-sensitive sites when interpreted with appropriate caution. Such sites may be especially informative when they occur near active-site entrances, subunit interfaces or symmetry-related junctions within oligomeric catalytic cores.

In the present study, we report the crystal structure of *Ec*DSCD in space group *F*432 and compare it with previously described *Ec*DSCD crystal forms to examine local structural features of the 24-mer catalytic core. The structure was obtained from an endogenous *E. coli* protein that unexpectedly copurified during purification of an unrelated target, but the resulting *F*432 crystal form captures *Ec*DSCD in a symmetry-expanded architecture that closely reflects the octahedral organization of the E2 catalytic core. Structural analysis revealed a compact trimeric building block, sulfate-compatible density at the intratrimeric junction and a different orientation of the α1-helix near the active-site pocket. Together, these features define a local structural arrangement that is particularly evident in the *F*432 crystal form.

## 2. Materials and methods

### 2.1. Protein expression and purification of copurified *Ec*DSCD

The *E. coli* dihydrolipoamide succinyltransferase catalytic domain (*Ec*DSCD) investigated in this study was serendipitously obtained as a copurified endogenous protein during the purification of a recombinant target protein. Specifically, *E. coli* BL21 (DE3) cells harboring a pET-21a (Novagen)-based recombinant plasmid were inoculated into LB medium containing 50 µg ml^−1^ ampicillin. The culture was grown at 37°C with shaking at 150 rev min^−1^ until the optical density at 600 nm (OD_600_) reached approximately 0.5. Protein expression was induced by adding isopropyl β-d-1-thiogalactopyranoside (IPTG) to a final concentration of 0.5 m*M*, followed by incubation for an additional 4 h at 37°C. The cells were harvested by centrifugation at 4500*g* for 10 min at 4°C. The resulting cell pellet was resuspended in a buffer consisting of 50 m*M* Tris–HCl pH 7.5, 500 m*M* NaCl. Cell lysis was performed by sonication on ice using a Cole–Parmer ultrasonic processor at 40% amplitude, with alternating sonication and rest intervals for a total processing time of 40 min. Insoluble debris was removed from the crude extract by centrifugation at 20 000*g* for 1 h at 4°C.

The supernatant was applied onto an Ni–NTA (Ni^2+^-nitrilotriacetic acid) agarose column (Qiagen; utilizing 3 ml bed volume for each litre of bacterial growth) that had previously been equilibrated. Unbound components were removed by passing ten column volumes of washing buffer (50 m*M* Tris–HCl pH 7.5, 500 m*M* NaCl) through the resin. To elute the bound proteins, we applied a recovery buffer consisting of 50 m*M* Tris–HCl pH 7.5, 500 m*M* NaCl, 100 m*M* imidazole. The eluted samples were examined by SDS–PAGE, with subsequent Coomassie Brilliant Blue staining. For subsequent purification and buffer substitution, the combined samples were applied onto a Superdex 75 10/300 GL gel-filtration setup (Cytiva). This size-exclusion step utilized a buffer consisting of 50 m*M* Tris–HCl pH 7.5, 150 m*M* NaCl. The major peak fractions containing the target protein, as judged by SDS–PAGE analysis, were pooled and concentrated to 7 mg ml^−1^ using an Amicon Ultra-15 centrifugal filter unit with a 10 000 Da molecular-weight cutoff (Millipore) for subsequent crystallization trials. The crystal structure subsequently revealed *Ec*DSCD as the copurified endogenous protein.

### 2.2. Biochemical reanalysis of the retained crystallization sample

To obtain biochemical evidence regarding the composition of the sample that produced the unexpected crystals, the retained crystallization sample was reanalyzed by SDS–PAGE and anti-His Western blotting. In parallel, a non-induced control *E. coli* culture was subjected to the same purification procedure to assess whether endogenous host proteins could copurify and generate peak-like size-exclusion chromatography (SEC) profiles within a similar elution-volume range. The control culture was grown without IPTG induction, harvested and lysed under the same conditions used for preparation of the crystallization sample. The clarified lysate was subjected to the same two-step purification procedure used for preparation of the crystallization sample. Firstly, the lysate was applied onto an Ni–NTA agarose column equilibrated with 50 m*M* Tris–HCl pH 7.5, 500 m*M* NaCl. After washing, bound proteins were eluted with buffer consisting of 50 m*M* Tris–HCl pH 7.5, 500 m*M* NaCl, 100 m*M* imidazole. The eluate was subsequently analyzed by SEC using a Superdex 75 10/300 GL column equilibrated with 50 m*M* Tris–HCl pH 7.5, 150 m*M* NaCl.

The control purification sample and the retained crystallization sample were analyzed by SDS–PAGE and anti-His Western blotting. Equivalent amounts of each sample were loaded onto a single 12.5% SDS–PAGE gel. Following electrophoresis, the gel was divided into two parts containing identical sample sets. One part was stained with Coomassie Brilliant Blue, whereas proteins separated on the other part were transferred onto a polyvinylidene difluoride (PVDF) membrane for Western blot analysis. The membrane was blocked for 2 h at room temperature in phosphate-buffered saline containing 0.1% Tween 20 (PBST) supplemented with 10% skimmed milk and 5%(*w*/*v*) BSA. The membrane was then incubated for 2 h at room temperature with a rabbit monoclonal anti-His-tag antibody (1:10 000; AE086; ABclonal) diluted in blocking buffer. After three 5 min washes with PBST, the membrane was incubated for 45 min at room temperature with HRP-conjugated goat anti-rabbit IgG (H+L) secondary antibody (1:5000; AS014; ABclonal). Following three additional 5 min washes with PBST, immunoreactive bands were detected using an enhanced chemiluminescence substrate and visualized with an Azure 600 imaging system (Azure Biosystems).

### 2.3. Crystallization, data collection and structural analysis

Crystallization trials were carried out at 20°C using the hanging-drop vapor-diffusion method in 48-well VDX plates (Hampton Research). For each trial, 1 µl protein solution was mixed with 1 µl precipitant solution and equilibrated against 250 µl reservoir solution. The optimized crystallization condition consisted of 1.26 *M* ammonium sulfate, 100 m*M* MES pH 6.0. Prior to data collection, crystals were cryoprotected by transferring them into mother liquor supplemented with 20%(*v*/*v*) glycerol for approximately 30–60 s and then flash-cooled in liquid nitrogen. X-ray diffraction data were collected at 100 K on beamline 7A at the Pohang Accelerator Laboratory (PAL). Diffraction images were indexed, integrated and scaled with the *HKL*-2000 program package (Otwinowski & Minor, 1997 ▸).

The crystal structure of the crystallized copurified protein, assigned as *Ec*DSCD, was determined by molecular replacement (MR). To identify the optimal search model for this copurified protein, the sequence-independent MR pipeline *SIMBAD*, implemented in the *CCP*4 suite, was employed (Winn *et al.*, 2011 ▸; Simpkin *et al.*, 2018 ▸; Agirre *et al.*, 2023 ▸). *SIMBAD* unambiguously identified *E. coli* DSCD (PDB entry 1scz; N. Schormann, J. Symersky, M. Carson, M. Luo, J. Tsao, D. Johnson, W.-Y. Huang, P. Pruett, G.Lin, S. Li, S. Qiu, A. Arabashi, B. Bunzel, D. Luo, L. Nagy, R. Gray, C.-H. Luan, Z. Zhang, S. Lu & L. DeLucas, unpublished work) as the top structural hit. Subsequently, targeted MR was performed using *Phaser*, with PDB entry 1scz as the search model (McCoy *et al.*, 2007 ▸). Model building was carried out through iterative cycles in *Coot*, followed by refinement with *REFMAC*5 and *phenix.refine* (Emsley & Cowtan, 2004 ▸; Murshudov *et al.*, 2011 ▸; Afonine *et al.*, 2012 ▸). For cross-validation, 5% of the diffraction data were excluded from refinement and used to calculate *R*_free_. Comparative refinements using 3.6, 3.5 and 3.4 Å cutoffs showed increased *R*_free_ values and *R*_free_–*R*_work_ gaps without meaningful improvement in model geometry; therefore, 3.6 Å was retained as the high-resolution cutoff. The final crystallographic statistics are summarized in Table 1 ▸. Structural alignments and molecular graphics were prepared using *PyMOL* (https://www.pymol.org).

### 2.4. Accession number

The protein coordinates and structure factors have been deposited in the RCSB Protein Data Bank under accession code 27nv. Residue numbers refer to the canonical *E. coli* SucB sequence numbering in UniProtKB (P0AFG6).

## 3. Results

### 3.1. Unexpected copurification and crystallization of *Ec*DSCD

The intended His-tagged target protein was purified in two independent batches and appeared to be highly enriched by SDS–PAGE. Crystals obtained from Batch 1 appeared within approximately two weeks, and structure determination confirmed that they contained the intended target protein. In contrast, Batch 2 showed minor additional bands on SDS–PAGE and displayed a more asymmetric SEC profile with a pronounced shoulder. Crystals from Batch 2 only appeared after more than four months of incubation. Anti-His Western blotting confirmed the presence of the intended His-tagged protein in the retained Batch 2 sample, whereas the additional Coomassie-stained protein species showed no detectable anti-His reactivity, consistent with the presence of copurified endogenous *E. coli* proteins. In parallel, a non-induced *E. coli* control subjected to SEC procedures produced peak-like signals within a similar elution-volume range. Together, these observations showed that endogenous host proteins could persist through the purification procedure and coexist with the intended target in the crystallization sample. This biochemical evidence supported the possibility that a copurified endogenous protein contributed to the unexpected crystallization outcome and was consistent with the subsequent crystallo­graphic assignment of the crystallized protein as *Ec*DSCD (Supplementary Fig. S1).

The late-appearing crystals were initially assumed to contain the intended target because of their similar morphology. However, diffraction analysis revealed a different space group, and molecular replacement using the target protein model failed to produce a valid solution, with *Phaser* TFZ values below 6 and the refinement *R*_free_ remaining above 50%. Because the unusual *F*432 symmetry raised the possibility that an unintended protein had crystallized, we used the sequence-independent *SIMBAD* molecular-replacement pipeline, which is designed to identify unexpected crystal contaminants (Simpkin *et al.*, 2018 ▸). In the lattice-parameter search, *SIMBAD* identified the previously reported *Ec*DSCD structure, PDB entry 1scz, as the only convincing solution. Without further optimization of the search model, a single *SIMBAD* run yielded a *Phaser* TFZ of 21.5 and an *R*_free_ of 29.3%. These results strongly supported the assignment of the crystallized protein as *Ec*DSCD. Following iterative model building and refinement, the *Ec*DSCD structure was refined to the statistics summarized in Table 1 ▸.

### 3.2. *F*432 crystal symmetry recapitulates the octahedral 24-mer architecture of *Ec*DSCD

**Figure 1:**
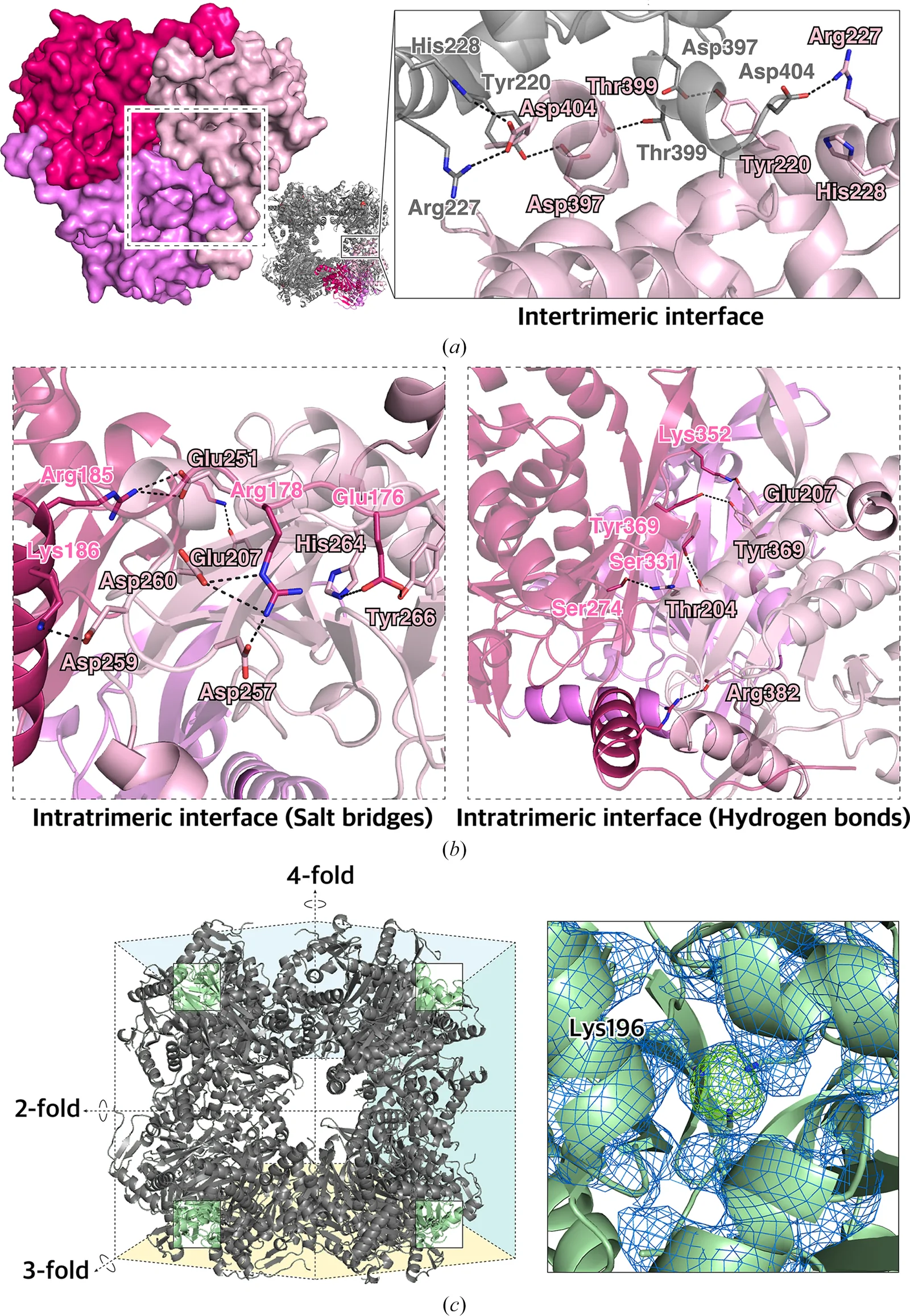
Structural organization of the *Ec*DSCD *F*432 assembly and sulfate-compatible density at the intratrimeric junction. (*a*) Surface representation of the symmetry-generated *Ec*DSCD trimer, showing the interlocked arrangement of the three protomers. A symmetry-expanded view illustrates how eight trimers assemble into the 24-mer particle. (*b*) Close-up views of the symmetry-related intratrimeric interface. Representative interprotomer contacts show that the trimer interface is supported by complementary packing together with ionic interaction networks and hydrogen bonds. The boxed region in (*a*) is enlarged in (*b*). (*c*) Symmetry-expanded *Ec*DSCD 24-mer assembly within the crystallographic unit cell, showing sulfate-compatible density near the Lys196-centered threefold intratrimeric junction. The 2*F*_o_ − *F*_c_ electron-density map is shown as a blue mesh contoured at 1.0σ, and the positive *F*_o_ − *F*_c_ difference map is shown as a green mesh contoured at 3.0σ. The sulfate ion was not modeled because symmetry-related copies would overlap at this position.

In the *F*432 crystal form, *Ec*DSCD protomers are related by crystallographic symmetry to form a compact trimer, and eight such trimers assemble into a 24-subunit particle with octa­hedral 432 symmetry. Here, intratrimeric interfaces refer to contacts between protomers within an *Ec*DSCD trimer, whereas intertrimeric interfaces refer to contacts between neighboring trimers in the symmetry-expanded 24-mer assembly. Surface representations show that the three protomers are not loosely associated, but instead form an interlocked trimer in which complementary protrusions and grooves allow tight protomer–protomer packing (Fig. 1 ▸*a*). Because the contacts are symmetry-generated, the three intratrimeric interfaces represent equivalent copies of one symmetry-unique interface. Approximately 170 N-terminal residues were not resolved. The local crystal packing around the modeled N-terminus appears insufficient to accommodate the entire missing segment as a fully ordered extension. However, this does not define the exact fragment boundary, as the unresolved region may reflect truncation, conformational disorder or both.

*PISA* analysis showed that the intratrimeric interface buries approximately 880 Å^2^ of solvent-accessible surface area per protomer–protomer contact and involves 45 and 38 residues from the opposing protomers (Krissinel & Henrick, 2007 ▸). The interface is supported by complementary packing together with hydrogen bonds and electrostatic interactions. Ionic contacts were mainly observed near the N-terminal/α1-helix region, whereas hydrogen bonds were distributed predominantly within the inner interface. Although the calculated solvation-energy gain for a single interface was moderate, ranging from −3.3 to −3.5 kcal mol^−1^, the repeated symmetry-equivalent contacts and pronounced shape complementarity support the formation of a compact trimeric building block in this crystal form (Fig. 1 ▸*b*).

In the symmetry-expanded 24-mer, neighboring trimers are connected through localized intertrimeric junctions to form a closed octahedral core. *PISA* analysis showed that this 24-mer-forming interface buries approximately 921 Å^2^ of solvent-accessible surface area, comparable to the intratrimeric interface, but with a more favorable solvation energy gain of −6.4 to −7.5 kcal mol^−1^. The junction is centered near the C-terminal region, where Asp404 forms polar/electrostatic contacts with Arg227 and His228 from the neighboring protomer, suggesting that C-terminal contacts help position adjacent trimers within the 24-mer particle (Fig. 1 ▸*a*). Thus, the *F*432 structure captures *Ec*DSCD as a hierarchical assembly in which compact trimers are organized into the octahedral 24-mer through complementary protein–protein interfaces.

### 3.3. Sulfate-compatible density marks an intratrimeric junction

The overall fold and assembly of our *Ec*DSCD structure are highly similar to previously reported *E. coli* DSCD structures. Among these structures, the *F*432 crystal form is notable because it reflects the octahedral 432 symmetry of the E2 catalytic core, in which eight trimers assemble into a 24-mer particle. Thus, the *F*432 packing observed in our structure likely represents the biologically relevant 24-mer architecture rather than an arbitrary crystal-packing arrangement.

In our *F*432 structure, a strong sulfate-compatible electron density was observed near Lys196 at the crystallographic threefold axis, where symmetry-related protomers converge. Because modeling a full-occupancy sulfate ion at this symmetry-related position would generate steric clashes among symmetry-equivalent copies, sulfate was not included in the final model. Instead, this density was interpreted as a partially occupied or symmetry-averaged tetrahedral oxy­anion site. Its location near Lys196 suggests that this region provides a positively charged environment capable of accommodating sulfate or sulfate-like oxyanions at the intratrimeric junction (Fig. 1 ▸*c*).

**Figure 2:**
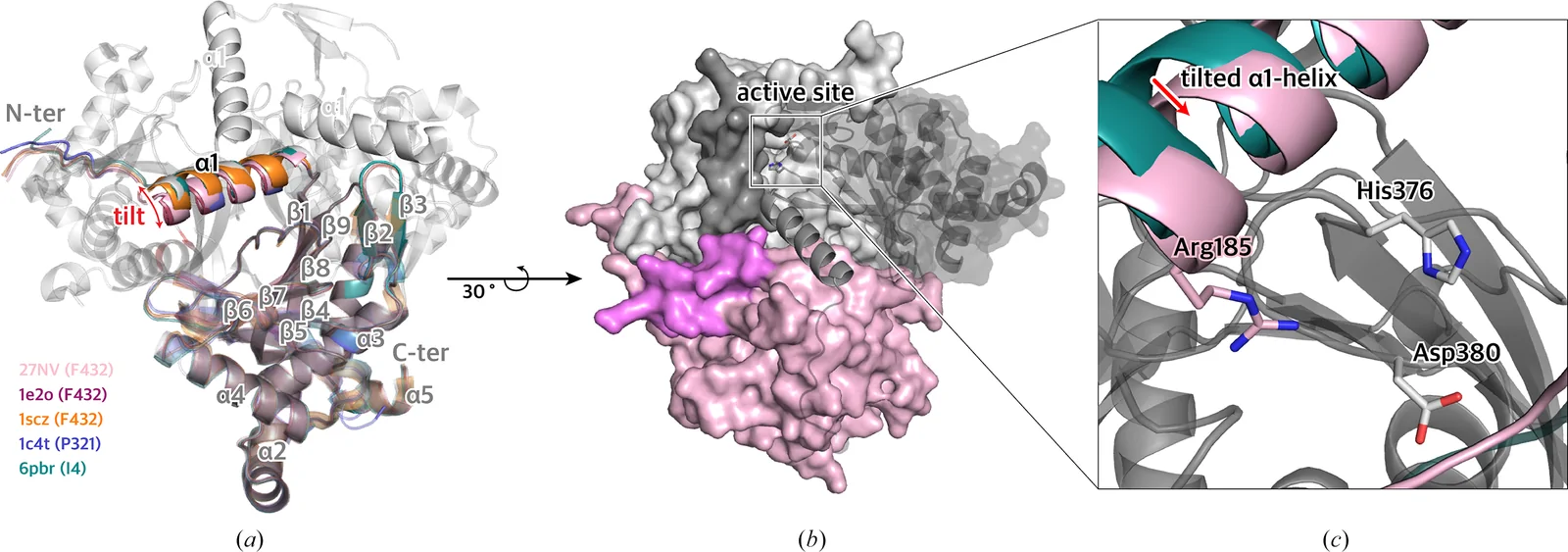
Positioning of the α1-helix near the active-site pocket of *Ec*DSCD. (*a*) Monomer superposition of the five available *Ec*DSCD structures showing the conserved topology but variable orientations of the α1-helix. (*b*) Surface representation of the *Ec*DSCD trimer showing the active-site pocket near the α1-helix region. (*c*) Close-up comparison of the α1-helix positions in the present structure and PDB entry 6pbr near the active-site entrance. In the present structure, the α1-helix lies adjacent to Arg185 and the catalytic residues His376 and Asp380, positioning it above the entrance to the active-site pocket.

Comparison with previously reported *Ec*DSCD structures supports this interpretation. The sulfate-containing *F*432 structure PDB entry 1e2o and our structure show sulfate-compatible density near the threefold axis, whereas the sulfate-free *F*432 structure PDB entry 1scz adopts the same overall cubic packing without an ordered sulfate ion. In addition, sulfate-associated density is also observed in PDB entry 1c4t, which was crystallized in the presence of sulfate but adopts a different crystal packing (Knapp *et al.*, 1998 ▸, 2000 ▸; Andi *et al.*, 2019 ▸). These comparisons suggest that sulfate is not required for formation of the *F*432 24-mer architecture, but may occupy a pre-existing oxyanion-sensitive site. Together, these observations indicate that the Lys196-centered sulfate-compatible density marks a local anion-associated hotspot at the *F*432 intratrimeric junction (Fig. 2 ▸*a* and Table 2 ▸).

### 3.4. The α1-helix connects the intratrimeric junction to the active-site pocket

Structural comparison of *Ec*DSCD crystal forms revealed that one of the most prominent local differences occurs in the α1-helix region. In our *F*432 structure, the α1-helix, corresponding to residues 183–198, is arranged around the crystallographic threefold axis in a pinwheel-like manner. This arrangement places the α1-helix adjacent to the oxyanion-associated intratrimeric junction described above, suggesting that this helix is structurally connected to the local organization of the 24-mer assembly.

The α1-helix is also positioned near the entrance of the catalytic-domain active-site pocket. In the current structure, interpretable electron density begins at residue 173, whereas the preceding N-terminal segment is not resolved. Because the exact terminal boundary is unknown, the absence of density cannot distinguish truncation from conformational disorder. Thus, the α1-helix lies at the N-terminal edge of the structured catalytic core, where it may contribute to shaping the active-site pocket (Fig. 2 ▸*a*).

Superposition with previously reported *Ec*DSCD structures showed that the α1-helix adopts slightly different angular orientations among the available structures. Relative to our *F*432 structure, the angular differences were 1.40° for PDB entry 1c4t, 1.86° for PDB entry 1e2o, 3.58° for PDB entry 1scz and 4.69° for PDB entry 6pbr. Thus, the α1-helix in our structure more closely resembles those in the sulfate-containing structures PDB entries 1c4t and 1e2o than those in PDB entries 1scz and 6pbr. However, given the limited number of available structures and their different crystallization conditions and packing environments, this apparent trend does not establish a sulfate-dependent conformational change. In our structure, the α1-helix is oriented towards the active-site pocket, producing a relatively closed or capped active-site-proximal arrangement. These comparisons suggest that α1-helix positioning may modulate the size and shape of the active-site entrance (Fig. 2 ▸*a* and Table 2 ▸).

The active-site region of *Ec*DSCD includes conserved catalytic residues His376 and Asp380, with nearby residues such as Thr324 contributing to the local pocket environment. Although the α1-helix does not directly constitute the catalytic center, its position allows it to influence the architecture of the active-site pocket from above, acting as a cap-like structural element (Knapp *et al.*, 1998 ▸; Chakraborty *et al.*, 2018 ▸). Together, these observations suggest that the α1-helix may provide a structural connection between the intratrimeric junction and the catalytic-domain active-site pocket (Figs. 2 ▸*b* and 2 ▸*c*).

## 4. Discussion

Endogenous *E. coli* proteins can occasionally co-purify with recombinant targets and crystallize unexpectedly when their electrophoretic and chromatographic properties overlap (Niedzialkowska *et al.*, 2016 ▸). In such cases, sequence-independent molecular-replacement approaches such as *SIMBAD* can identify the crystallized protein when conventional molecular replacement with the intended target fails. Although our crystallographic analysis supports assignment of the protein as an *Ec*DSCD catalytic-domain fragment, its exact terminal boundaries and the mechanism of fragment formation remain uncertain.

The *Ec*DSCD catalytic domain transfers the succinyl group from dihydrolipoamide to CoA, producing succinyl-CoA. CoA is therefore an essential substrate, and its negatively charged phosphate-containing region contributes to recognition and positioning near the catalytic pocket. Sulfate and phosphate are not functionally equivalent, but they share tetrahedral geometry and anionic character and can occupy similar electropositive environments in protein structures. The sulfate-compatible density near Lys196 does not coincide exactly with the canonical CoA-binding pocket, but lies near its entrance in a region that could be approached by the phosphate-containing portion of CoA. This site may therefore represent an electrostatic environment relevant to phosphate recognition rather than a mimic of the entire CoA molecule.

Comparison of the available *Ec*DSCD structures illustrates how crystallography can reveal subtle conformational variability despite highly conserved overall folds. The observed differences in sulfate-associated density and α1-helix orientation are modest, but suggest a possible relationship between local anion-sensitive interactions and the geometry of the active-site entrance. The limited number of structures and differences in crystallization conditions prevent a definitive mechanistic conclusion. Nevertheless, these structural details provide testable hypotheses regarding CoA recognition and the still poorly understood functional role of the α1-helix.

Overall, the present structure links the intratrimeric junction, a Lys196-centered oxyanion-sensitive region, and α1-helix positioning near the active-site entrance. Further structural and biochemical studies will be required to determine whether oxyanion occupancy directly influences α1-helix orientation or CoA access.

## Supplementary Material

PDB reference: catalytic domain of *E. coli* dihydrolipoamide succinyltransferase, 27nv

Supplementary Figure S1. DOI: 10.1107/S2053230X2600909X/ft5129sup1.pdf

## Figures and Tables

**Table 1:** Crystallographic data-collection and refinement statistics for *Ec*DSCD Values in parentheses are for the highest resolution shell.

Data collection	
Beamline	PAL 7A
Wavelength (Å)	0.97
Resolution range (Å)	38.8–3.6
Space group	*F*432
*a*, *b*, *c* (Å)	219.8, 219.8, 219.8
α, β, γ (°)	90, 90, 90
Completeness (%)	100 (100)
*R*_merge_ (%)	44.6 (135.2)
CC_1/2_	0.98 (0.89)
Multiplicity	16 (14.6)
〈*I*/σ(*I*)〉	19.3 (10.0)
Refinement	
*R*_work_†/*R*_free_† (%)	21.7/25.0
No. of atoms	1819
Average *B* value (Å^2^)	115.0
R.m.s.d., bond lengths (Å)	0.007
R.m.s.d., angles (°)	1.805

† *R*_work_ = 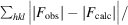

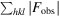
, where *F*_obs_ is the observed structure-factor amplitude and *F*_calc_ is the structure factor calculated from the model. *R*_free_ is computed in the same manner as *R*_work_, but from a test set containing 5% of data excluded from the refinement calculation.

**Table 2:** Comparison of representative *Ec*DSCD crystal structures Comparison of crystal forms, crystallization conditions, sulfate-compatible density and structural features of *Ec*DSCD crystal structures. Δα1-helix angles were calculated relative to the present structure after monomer superposition.

PDB code	Space group	Sulfate-compatible density	Crystallization condition	Resolution (Å)	Assembly-related note	Δ α1-helix angle (°)
1e2o	*F*432	Yes	Ammonium sulfate-containing	3.0	N-terminally His-tagged *Ec*DSCD	1.86
1scz	*F*432	No	PEG/ammonium acetate/magnesium acetate	2.2	Sulfate-free *F*432 form	3.58
1c4t	*P*3_1_21	Yes	Ammonium sulfate-containing	3.0	C-terminally His-tagged; altered packing	1.40
6pbr	*I*4	No	High-salt NaCl condition	3.0	Tag-free contaminant structure	4.69
This work	*F*432	Yes	Ammonium sulfate-containing	3.6	Fortuitous *Ec*DSCD crystal	—

## Data Availability

The atomic coordinates and structure factors have been deposited in the RCSB Protein Data Bank under accession code 27nv.
