# Supplementary material for: Sulfate-compatible density marks an intratrimeric junction in the Escherichia coli dihydrolipoamide succinyltransferase catalytic domain
Source: Acta Crystallogr F Struct Biol Commun. 2026 Sep 17;82(Pt 10):362–8. doi: 10.1107/S2053230X2600909X (PMC13629643; doi:10.1107/S2053230X2600909X)
Supplement: Supplementary file 1 [file f-82-00362-sup1.pdf]

STRUCTURAL BIOLOGY COMMUNICATIONS

Volume 82 (2026)

Supporting information for article:

Sulfate-compatible density marks an intratrimeric junction in the *Escherichia coli* dihydrolipoamide succinyltransferase catalytic domain

Bison Lim, Eunji Kim, Jungeun Byun, Minjeong Kim and Hyo Jung Kim

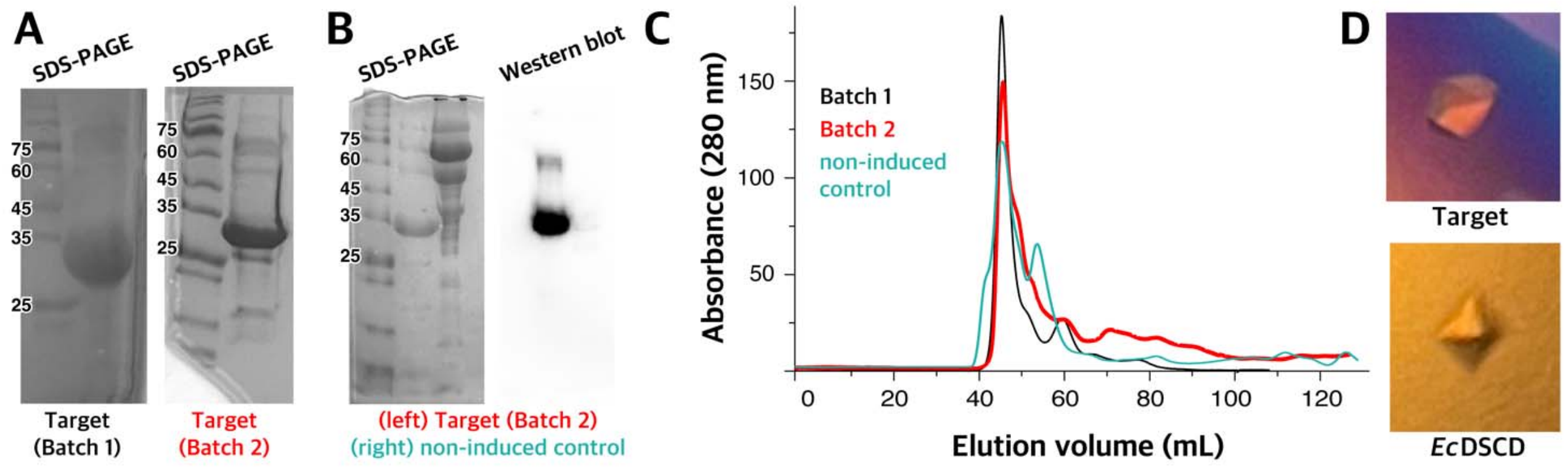

Figure S1 Biochemical reanalysis of the retained crystallization sample and control purification. (A) Coomassie-stained SDS-PAGE analysis of purification Batch 1 and Batch 2. Both preparations contained a prominent protein band in the 20-30 kDa region, whereas additional lower-intensity bands were evident in Batch 2, indicating greater sample heterogeneity. (B) Coomassie-stained SDS-PAGE and anti-His Western blot analysis of the retained crystallization sample and the non-induced control purification sample. The prominent band in the retained crystallization sample was reactive with the anti-His antibody, consistent with the presence of the intended His-tagged target. Additional Coomassie-stained protein species were not detected by the anti-His antibody. The non-induced control also contained endogenous proteins that persisted through the purification procedure but showed no detectable anti-His reactivity. (C) Size-exclusion chromatography profiles of Batch 1, Batch 2, and the non-induced control purification sample. Batch 2 displayed a more asymmetric elution profile with a pronounced shoulder, suggesting increased sample heterogeneity relative to Batch 1. The control sample also produced peak-like signals within a similar elution-volume range, demonstrating that endogenous *E. coli* proteins could persist through the Ni-NTA and size-exclusion chromatography procedures. (D) Representative crystals obtained from Batch 1 and Batch 2. Batch 1 yielded the intended target, whereas the Batch 2 crystals were assigned as *Ec*DSCD by crystallographic analysis. Despite their different protein identities, the crystals showed similar macroscopic morphologies.
